# Exploring Macroporosity of Additively Manufactured Titanium Metamaterials for Bone Regeneration with Quality by Design: A Systematic Literature Review

**DOI:** 10.3390/ma13214794

**Published:** 2020-10-27

**Authors:** Daniel Martinez-Marquez, Ylva Delmar, Shoujin Sun, Rodney A. Stewart

**Affiliations:** School of Engineering and Built Environment, Griffith University, Gold Coast, QLD 4222, Australia; daniel.martinezmarquez@griffithuni.edu.au (D.M.-M.); ylvadelmar@hotmail.com (Y.D.); shoujin.sun@griffith.edu.au (S.S.)

**Keywords:** porous implants, bone implants, metamaterials, titanium, mechanical properties, pore size, unit cell, porosity, elastic modulus, compressive strength, additive manufacturing

## Abstract

Additive manufacturing facilitates the design of porous metal implants with detailed internal architecture. A rationally designed porous structure can provide to biocompatible titanium alloys biomimetic mechanical and biological properties for bone regeneration. However, increased porosity results in decreased material strength. The porosity and pore sizes that are ideal for porous implants are still controversial in the literature, complicating the justification of a design decision. Recently, metallic porous biomaterials have been proposed for load-bearing applications beyond surface coatings. This recent science lacks standards, but the Quality by Design (QbD) system can assist the design process in a systematic way. This study used the QbD system to explore the Quality Target Product Profile and Ideal Quality Attributes of additively manufactured titanium porous scaffolds for bone regeneration with a biomimetic approach. For this purpose, a total of 807 experimental results extracted from 50 different studies were benchmarked against proposed target values based on bone properties, governmental regulations, and scientific research relevant to bone implants. The scaffold properties such as unit cell geometry, pore size, porosity, compressive strength, and fatigue strength were studied. The results of this study may help future research to effectively direct the design process under the QbD system.

## 1. Introduction

### 1.1. Current Issues with Traditional Bone Implants and Scaffolds

Many physical conditions necessitate bone tissue replacements and joint implants. Some of these conditions are caused by degenerative diseases, birth defects, and orthopaedic traumas [1]. However, despite the tremendous progress in biomedical engineering, 20% of patients subjected to joint reconstructive surgery experience significant problems [2]. This situation is reflected in the fact that orthopaedic products, such as knee and hip prostheses, are the fifth most recalled medical products; of these recalls, 48% are due to manufacturing issues and 34% to design flaws [3,4]. Some of the main flaws with orthopaedic implants are associated with their longevity, material properties, and mismatch with patient size and shape requirements [5,6]. Stress shielding is one of the main design flaws of load-bearing prostheses. This phenomenon occurs because bone is a self-healing material that requires load application to remodel itself, but a material with a higher modulus of elasticity (*E*) absorbs all the stress generated, leading to bone reabsorption and subsequent loosening of the implant [7].

In the case of bone defects, they can be caused by tumour resection, infections, complex fractures, and non-unions [8]. The most common treatment for bone defects is surgical intervention, where an autograft (bone taken from the patient’s body) is used to fill bone defect spaces [9]. However, due to their restricted availability, allografts (bone tissue from a deceased donor) are frequently used to treat critical-size defects [9]. Bone grafting is a common surgical procedure; it has been estimated that 2.2 million grafting procedures are performed worldwide each year [8]. However, late graft rupture has been reported to be as high as 60% 10 years after the grafting procedure [10]. Allograft transplantation has a success rate of approximately 70%. The low success rate of allografts is caused by the prevalence of infection, rejection by the host’s immune system, fatigue fractures, delayed union, non-union, and incomplete graft resorption [11,12]. In the case of autografts, the disadvantages are increased post-operative morbidity, lack of available tissue, chronic pain, infection, nerve injury, and weakened bone donor graft sites [12,13].

To solve these grafting problems, several scaffold traditional techniques have been used without much success: solvent-casting particulate-leaching, gas foaming, fibre meshes (fibre bonding), phase separation, melt moulding, emulsion freeze drying, solution casting, and freeze drying [14]. Some of the disadvantages of traditional scaffold fabrication techniques are their lack of control over porosity characteristics, such as pore size, pore distribution, and interconnectivity; the toxic by-products of scaffold degradation; and their lack of consistent mechanical properties [14]. Hence, traditional techniques for bone reconstruction including grafting and prostheses are not sufficiently effective, which represent a medical challenge that comes with several limitations and risks [9]. Moreover, no material yet exists with the ideal properties for bone tissue replacement [15,16,17]. To overcome these issues, tissue engineering has focused on additive manufacturing technologies to produce the next generation of bone implants and scaffolds.

### 1.2. Additive Manufacturing

Additive manufacturing (AM) technologies, supported by computer-aided design (CAD) software, progressively build 3D physical objects from a series of cross-sections, which are joined together to create a final shape [18]. With AM, it is possible to create complex interconnected and porous structures with controlled pore size, shape, and distribution and properties resembling bone mechanical properties, such as a modulus of elasticity to induce bone ingrowth [19,20]. This capability permits the fabrication of hierarchical structures at the microscale and the manipulation of material properties to create metamaterials. In terms of implant design, this advance means that products can be designed with a biomimetic approach according to the patient’s anatomy and the bone tissue’s mechanical properties [21]. The design freedom of AM allows its use in difficult clinical scenarios in which bone diseases, deformities, and trauma usually necessitate the reconstruction of bone defects with complex anatomical shapes, which is extremely difficult even for the most skilled surgeon [22]. The complex reconstruction of bone defects is possible through combining the advantages of AM with CAD and medical imaging technologies, such as computed tomography and magnetic resonance, to fabricate implants according to the patient’s specific anatomy, thus achieving an exact adaptation to the region of implantation [23]. In the search of suitable materials for AM, bone regeneration, and implant application tissue engineering has focused on developing a variety of different types of synthetic and natural materials.

### 1.3. Materials for Bone Regeneration and Implant Applications

Materials appropriate for implantation within the human body require distinct biocompatible properties. Therefore, in the selection of appropriate materials for implant applications, several factors must be considered. First, the intended implant location must be considered to predict host response, which is governed by the biochemical and physical environments in contact with the medical device [24,25]. Second, the material should possess appropriate biological and mechanical properties for its specific purpose to prevent physical damage to the body. Third, from the perspective of tissue engineering, materials should mimic one or multiple characteristics of the natural region of repair. In the case of bone repair, the desired characteristics are osteoconductivity, osteoinductivity, and osseointegration. As a result, for an optimum scaffold and prosthesis design, material science may combine several technologies to create suitable materials that fulfil these needs.

#### 1.3.1. Polymers

Polymers for AM and tissue engineering applications are biocompatible materials that offer several advantages over other materials, including biodegradability, cytocompatibility, easy processability, and flexibility in the tailoring of their properties [26]. Polymers can be classified as natural or synthetic and some of them already have regulatory approval [27].

Natural polymers are made from proteins such as alginate, gelatine, collagen, silk, chitosan, cellulose, and hyaluronic acid [28]. The advantages of natural polymers are their excellent biodegradability, low production costs, and superior chemical versatility, as well as their improved biological performance that allow better interactions with cells than other biomaterials, improving their attachment and differentiation [29]. However, natural polymers can be expensive to produce, due to the difficulty in controlling their mechanical properties, biodegradation rate, and quality consistency [30].

Due to the disadvantages of natural polymers, different synthetic polymers, such as polycaprolactone (PCL), polylactic acid (PLA), and poly Lactic-co-Glycolic Acid (PLGA), have been developed. Their advantages include low immunogenic potential, large scale low production cost, and good quality consistency [31]. Moreover, their mechanical properties, microstructure, and degradation rate can be tuned according to needs [27]. Despite the advantages of natural and synthetic polymers, they are unsuitable for load-bearing applications due to their lower modulus of elasticity compared to bone, unstable mechanical strength, and tendency to creep [32,33]. Hence, in recent years, a variety of polymers have been combined with different materials to such as bioceramics (e.g., bioglasses, tri-calcium phosphates, and carbon nanotubes) and metals to create composite materials with tunable mechanical properties as well as with the capacity to deliver drugs, exosomes, and growth factors, to name a few [34,35,36,37].

#### 1.3.2. Bioceramics

Bioceramics are a large group of materials used for bone substitution and regeneration. Calcium phosphate (CaP) ceramics is one of the main groups of bioceramics. Calcium phosphate ceramics are abundant in bone, constituting between 80% and 90% of bone’s anorganic matter. This group of bioceramics is widely used as implant coating, bone grafting, and more recently have been fabricated for bone scaffolding applications with AM [38,39]. Hydroxyapatite HAP and β-tricalcium phosphate (β-TCP), are the most-studied CaP bioceramics. The main advantages of calcium phosphate materials are their osteoinductive and osteoconductive properties, as well as their dissolution in body fluids [40]. For load-bearing applications, the major disadvantage of CaPs is their poor mechanical properties. Despite their good compressive strength, CaPs lack plastic deformation, making them brittle and prone to cracking. Consequently, these materials are not yet suitable for load-bearing applications [41]. Nevertheless, the lower wear rate of CaPs makes these materials the preferred choice for surface coating to reduce wear in joint prostheses [42]. They are also commonly used for spinal fusion, maxillofacial and cranio-maxillofacial reconstruction, as well as bone filler and bone cement due to their excellent biocompatibility and osteoconductivity [43].

Discovered in 1969 by Larry Hench, bioglasses are ceramic materials composed of calcium, phosphorus, and silicon dioxide [16]. Bioglasses are bioactive ceramic materials with strong osteointegrative and osteoconductive properties, as well as higher mechanical strength than calcium phosphate ceramics [44]. Hence, bioglasses have been intensely investigated with AM for bone tissue engineering applications [45,46]. The advantage of these materials is that by changing the proportions of their basic components, different forms with different properties can be obtained; for example, non-resorbable bioglasses can be transformed into resorbable bioglasses [44]. Moreover, they can be designed with controlled biodegradability and drug and cell delivery capabilities [47,48]. Their applications also include bioglass scaffolds produced using AM with controlled porosity architecture and improved mechanical properties for bone regeneration [49]. However, bioglasses are limited for use in practical load-bearing applications due to their low resistance to cyclic loading and their brittleness [50].

#### 1.3.3. Metals and Titanium as a Bio-Metamaterial

Metals have been the common choice to replace hard tissue in load-bearing applications due to their mechanical properties, corrosion resistance, and biocompatibility. Most of these materials are alloys, such as 316L stainless steel (316LSS), cobalt chromium (Co–Cr), and titanium (Ti) alloys [5]. Among all metallic materials, the titanium alloy Ti–6Al–4V is the gold standard for orthopaedic applications [51,52] because of its high biocompatibility [53], high corrosion resistance, low modulus of elasticity [5], and high strength-to-weight ratio [19]. Furthermore, Ti is a reactive metal that naturally forms a thin layer of oxide, which blocks metal ions from reaching its surface, increasing its biocompatibility [54]. The biomedical applications of Ti–6Al–4V are quite broad and encompass dental implants; hip, shoulder, knee, spine, elbow, and wrist replacements; bone fixation components; and cardiovascular applications [5].

Nevertheless, the most common problems of metallic materials are wear and the stress-shielding effect caused by their high modulus of elasticity compared with bone [52,55,56,57]. Moreover, despite the excellent biocompatibility and mechanical properties of Ti and Ti alloys, they usually require long healing periods to create a stable interface with the surrounding bone [58], with insufficient implant osseointegration as a potential outcome [59]. Hence, to further augment the biological, mass transport, and mechanical performance of Ti and Ti alloys different metamaterials have been developed. For example, metallic bone implants with a modulus of elasticity similar to that of bone can drastically reduce wear, shear stress, and bone resorption and consequently prevent implant loosening and revision surgery [60]. This may translate into enhanced quality of life for the patient, reductions in hospital expenses and recovery time, and improvement in joint dynamic performance [61]. With porous Ti and Ti alloy bio-metamaterials, osseointegration is also improved, and superior results have been accomplished in relation to mechanical properties. Nonetheless, pores act as stress concentrators, reducing the material load capacity [23]. As a result, for the design of load-bearing prostheses, it is crucial to balance mechanical properties with biological stimulation. Consequently, there have been several efforts to find the optimal balance between pore size and porosity percentage in different materials. For example, Zaharin et al. [62] investigated the effect of pore variation on the porosity and mechanical properties of several Ti–6A–l4V porous scaffolds. According to their results, scaffolds based on cube and gyroid unit cells with a pore size of 300 µm provided similar properties to bone. Moreover, they concluded that increments in porosity decreased the scaffolds’ elastic modulus and yield strength. In an earlier study Bobyn at al. [63] investigated the effects of pore size variation of cobalt-base alloy implants on the rate of bone growth. For this purpose, casted cobalt-base alloy implants were coated with powder particles and implanted into canine femurs for several weeks. The results indicated that pore sizes between 50 and 400 µm provided the maximum bone ingrowth and fixation strength.

Despite the excellent biocompatibility and mechanical properties of Ti and Ti alloys, they usually require long healing periods to create a stable interface with the surrounding bone, frequently resulting in insufficient osseointegration [64]. Hence, to further augment Ti’s bioactivity, corrosion resistance, and mechanical properties different mechanical, chemical, and physical surface modification methods have been developed [65,66,67]. Depending on the surface treatment used to modify Ti substrate, different topographic features can be achieved at the macroscale, microscale, and nanoscale. There is a large amount of evidence that rough Ti surfaces with topographic microfeatures better protein adsorption and provide higher osteoblasts attachment growth proliferation and activity than surface smooth surfaces [68]. Nonetheless, it has been demonstrated that nanoscale topography outperforms macro and micro-scale surface features towards augmenting cellular functions [69]. More recently, at has been proposed that a combination of different topographic features at the macro, micro, and nanoscale with local drug delivery functions can further enhance the biological, chemical, tribological, and mechanical performance of Ti bone implants [70,71,72,73].

### 1.4. Purpose and Objectives

**Figure 1:**
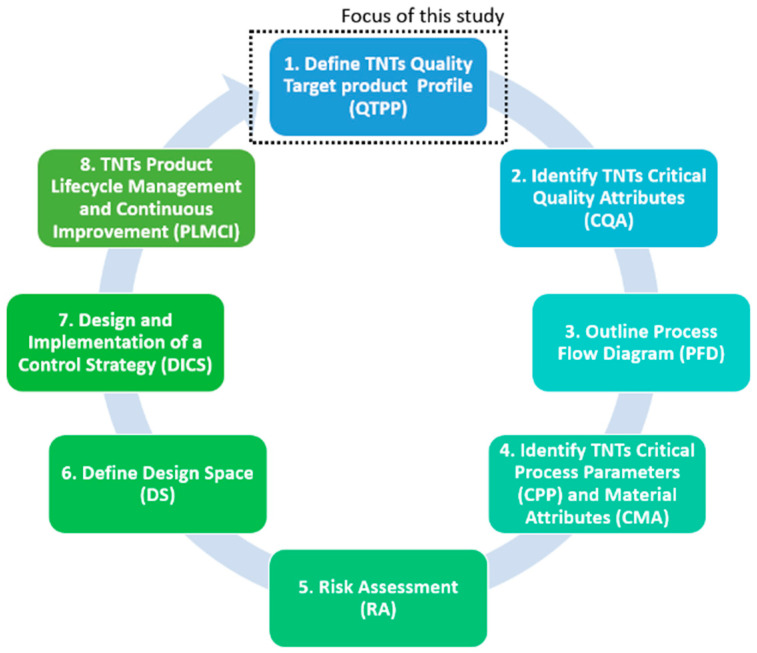
Schematic showing the focus of this study within the QbD system.

The purpose of this research is to provide researchers and industry with an in-depth adaptation of the Quality by Design (QbD) system for the fabrication of additively manufactured porous Ti implants considering the QbD guidelines for 3D printed bone implants and scaffolds [74]. The QbD system is composed by eight main steps that need to be systematically followed to acquire a complete comprehension of the product and its manufacturing process, including the identification and control of all variables to achieve the desired quality. Specifically, the scope of this present study was limited to the first step of the QbD framework (Figure 1). Thus, the objectives of this study are:

1. Define the ideal mechanical, geometrical and dimensional characteristics of the internal architecture of Ti bone scaffolds from a biomimetic perspective.
2. Compare the results of different studies on fully porous Ti structures in relation to the ideal quality attributes of bone scaffolds.
3. Identify the studies on fully porous Ti structures that satisfies the critical quality attributes of Ti porous bone implants and scaffolds.

## 2. Materials and Methods

This systematic research study is part of the implementation of the QbD approach for porous metal implants. Therefore, a constructive research approach was used to further extend the QbD system for patient-specific bone implants and scaffolds produced by AM [75,76]. For an in-depth interpretation and synthesis the researchers immersed themselves in the contextual literature [77]. This was an exploratory qualitative study which requires the collection of secondary data from various datasets of peer-reviewed publications following the Preferred Reporting Items for Systematic Reviews and Meta-Analyses (PRISMA) statement [78].

### 2.1. Data Collection

A systematic search was conducted on 20 January 2020 in the Science Direct and Google Scholar databases according to objectives and the PRISMA statement. Relevant keywords were connected with the Boolean operators “OR” and “AND”. Terms relevant to this research included the following: Titanium, Ti, additive manufacturing, 3D printing, rapid prototyping, bone tissue engineering, bone implant, implant, scaffold, prostheses, porous, porosity, and mechanical properties. To specify the search further, the terms were connected with Boolean operators (AND, OR): Implant(s); scaffold(s); prosthes(is, es); titanium; Ti; additive manufacturing; additively manufactured; 3D printing; and 3D printed.

The full phrase used was: (Implant* OR scaffold* OR prosthes?s) AND (titanium OR Ti) AND (“additive* manufactur*” OR “3D print*”) AND “mechanical properties” AND (porous OR porosity) AND “pore size” AND “elastic modulus” AND “fatigue”.

### 2.2. Study Selection

**Figure 2:**
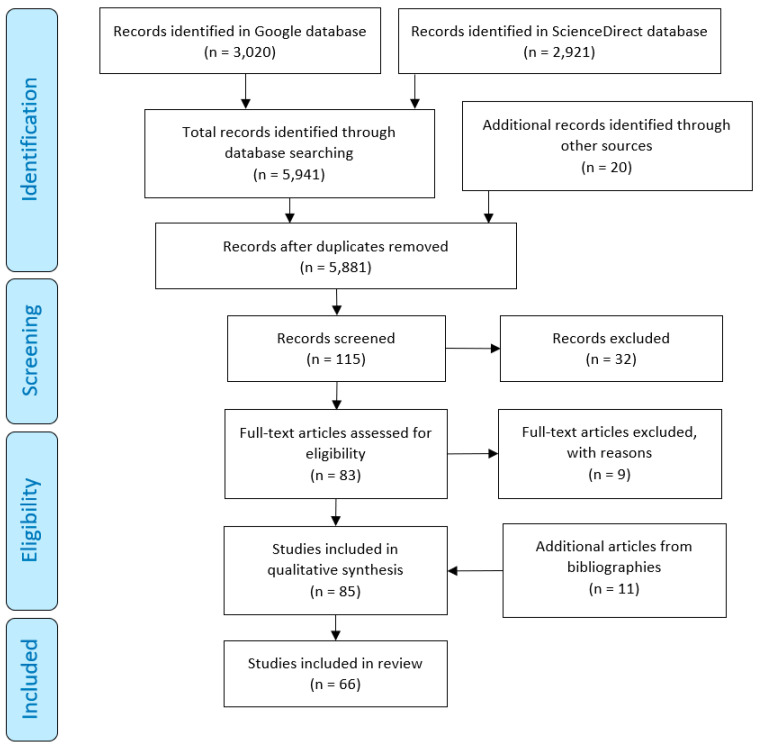
Search strategy and selection of studies in the Google Scholar and ScienceDirect databases.

Selected studies in the systematic literature search were limited to the following inclusion criteria: (1) peer-reviewed papers with full-text published within the last 20 years (2000 ± 2020); (2) empirical studies reporting the mechanical properties of Ti and Ti alloy porous scaffolds produced by AM for bone repair; (3) published in the English language; (4) the first 10 pages of the search results were assessed; and (5) the search results were sorted by relevance. From the systematic literature search in both the Science Direct and Google Scholar databases, a total of 5941 results were generated from which 83 articles were fully assessed, as presented in Table 1 and Figure 2.

### 2.3. Data Extraction and Analysis

The systematic literature search conducted in this study aimed to gather results of different studies regarding the characteristics of natural bone tissue, as well as the mechanical, geometrical, and dimensional properties of additively manufactured Ti porous implants with controlled porosity and/or pore size. The classification topics used in this study were pore size, pore shape, porosity, interconnectivity, multi-scaled, elastic modulus, compressive yield strength (σ_y_), ultimate compressive strength (σ_tu_), and fatigue strength. The references from the collected articles were systematically reviewed to identify further articles relevant to the subject. A full-text screening was performed by Y.D and D.M to avoid potential bias. A consensus meeting resolved any discrepancies between the reviewers.

Once all applicable literature had been identified, the extracted data were used to further extend the first step of the QbD system for fully porous Ti bone implants. Moreover, the data were also used to compare the different characteristics of Ti porous bone implants with natural bone tissue, and also to identify the most relevant characteristics that need to be imitated in the development of fully porous Ti bone implants.

## 3. Results

The systematic search identified a total of 64 different studies with data relevant to additively manufactured (AMd) porous Ti implants and scaffolds, as presented in Table 2 and Table 3. From the 64 studies identified, 14 studies were used to extract information related to bone structure and mechanical properties (Table 2). The remaining 50 studies provided detailed information in relation to different characteristics of porous Ti scaffolds fabricated by AM for bone implant purposes. A total of 807 experimental data from these studies was extracted, analysed, and categorised in the following categories: pore size, pore shape, porosity, multi-scaled porosity, elastic modulus, interconnectivity, yield strength, ultimate compressive strength, and fatigue strength, as shown in Table 3. However, from these nine categories multi-scaled porosity was excluded from the analysis due to insufficient data for further analysis. Therefore, a total of eight different implant features were selected due to availability of recorded data in scientific research articles. The selected eight features are: unit cell, porosity, pore size, interconnectivity, elastic modulus, compressive yield strength, ultimate compressive strength, and fatigue strength.

Through the systematic research performed in this study and by reviewing the medical device regulations from the Food and Drug Administration (FDA), a Quality Target Product Profile has been established including proposed values for the Ideal Quality Attributes for mechanical and dimensional properties of porous bone implants. These target properties are aimed for porous metal implant structures designed for load bearing implant applications. Following this, the results of the selected studies were compared and discussed, from a biomimetic point of view, with the characteristics of natural human bone to identify implants with properties similar or superior to human bone and current medical standards.

### 3.1. QbD Step 1: Ideal Quality Target Product Profile

The Quality Target Product Profile (QTPP) is critical for formulating the ideal features of a product considering both performance and safety. To direct the product development process, it is vital to understand user needs. Using QTPP, design failures can be identified early in the product development process to reduce costs and time. According to Martinez-Marquez et al. [36], the quality of bone implants should be defined from three perspectives: product-based, manufacturing-based, and user-based. In the context of fully porous Ti microstructures, the dimensions of quality considered most relevant are performance, features, reliability, conformance, durability, and perceived quality. The requirements relating to bone implants corresponding to these quality dimensions have been identified through studies of existing scientific research results.

### 3.2. Bone Implant Requirements

An additively manufactured bone implant must act as a stable scaffold that is biocompatible without causing inflammation or leaching material toxins into surrounding tissue. It requires a suitable surface that promotes cell adhesion and differentiation as well as provides a constant flow of cell nutrients and metabolic waste. This allows for bone tissue formation [81]. The scaffold material must have mechanical properties matching those of the surrounding tissues to avoid stress shielding and mechanical failure [81,82]. The bone pore size, geometry, interconnectivity, and porosity are microscopic features that make for the foundation of bone regeneration, cell growth, osteoconduction, and cell proliferation [74]. Designing implants with adequate pore dimensions allows for a constant flow of cell nutrients and waste. It also allows for sufficient connections to establish between the local bone area and the scaffold [74]. If the right attributes are chosen for the implant microstructure, it can mimic human bone’s natural characteristics, which is the end goal of biomimetic implant design [79,80]. Vasireddi and Basu [140] completed a list of general requirements for 3D printed implants:

- “A 3D, highly porous structure to support cell attachment, proliferation and extracellular matrix production;
- An interconnected pore network to promote oxygen, nutrient and waste exchange;
- A biocompatible and bioresorbable substrate with suitable degradation rates;
- An appropriate surface chemistry for cell attachment, proliferation and differentiation;
- Mechanical properties to support, or match, those of the tissues at the site of implantation; an architecture which promotes formation of the native anisotropic tissue structure; and
- An adapted geometry of clinically relevant size and shape.”

In similar research, Jabir et al. [141] described the main fundamental requirements for implants as biocompatibility, good manufacturability, geometric precision, appropriate design, biomechanical stability, resistance to implant wear, corrosion and aseptic loosening, bioactivity, and osteoconduction. Since the environment in the human body is highly corrosive and biomaterials are usually bioactive, the implant will interact with its environment after implantation [140]. The implant must therefore be designed with both useful functions and biological safety, providing utmost biocompatibility. The implant design must have a high degree of reproducibility, which will ensure faster and cheaper manufacturing as well as predictability and reliability.

Furthermore, the implant must be durable and of initial strength for safe handling during sterilisation, transport, and surgery, and to survive physical forces in vivo after implantation [142]. The implant will be subjected to constant load in the body, from walking and further strenuous movements [127]. Its mechanical strength is vital for its viability, where the implant must last the entirety of its expected lifetime without defects or failure [127].

Taking all the above into consideration in conjunction with the ideal eight quality dimensions of AMd bone implants proposed by Martinez-Marquez at al. [74], we proposed seven ideal quality dimensions of porous internal architecture of Ti bone implants. These quality dimensions are based on three quality perspectives, namely product, manufacturing, and user-based, as presented in Table 4.

### 3.3. QbD Step 1.1: Ideal Quality Attributes

The Ideal Quality Attributes (IQA) are the tissue or biological construct characteristics that must be mimicked to imitate the desired tissue biological architecture and functions. The IQA can be dimensional, physicochemical, mechanical, biological, or functional. However, if any technological and regulatory limitations exist it is important to consider that these IQA serve just as ‘ideal models’ to imitate even if it is not possible to achieve them. Therefore, the IQA can provide an ideal goal for the product development process in any tissue engineering project.

By studying the properties of natural human bone, we can find the different IQAs for bone implants [79,80]. As mentioned in Section 3.1, the implant must be of adequate strength while the elastic modulus must be similar to that of surrounding tissue to avoid damage [81]. The structure must be porous, using unit cells that allow for fluid movement and bone ingrowth, and the right porosity will reduce the implant stiffness. Interconnectivity completes the flow within the structure and facilitates bone ingrowth [82]. A combination of cancellous and cortical bone properties applied on bone implants can allow the implant to function like a natural bone, yet stronger without impairing surrounding native tissue. Another specific property of bone is its ability to heal itself when fractured [90,143]. The process involves cell migration, differentiation, and cellular proliferation [125]. The ability of natural bone to selfheal must be considered when designing a Ti implant, since the structure will not be able to do the same.

#### 3.3.1. Structure and Composition of Bone

**Figure 3:**
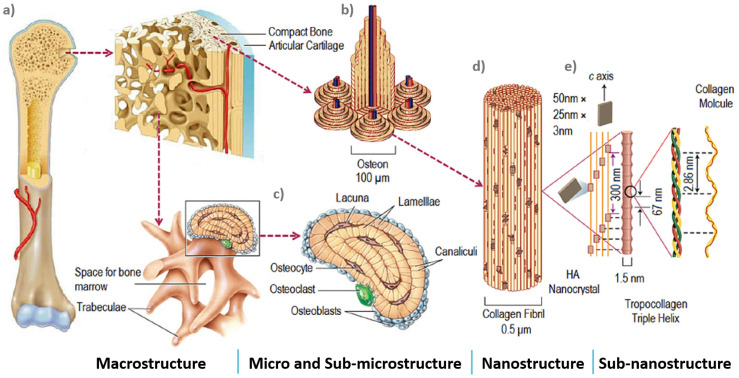
Hierarchical structural organization of bone: (**a**) cortical and cancellous bone; (**b**) osteons with Haversian systems; (**c**) lamellae; (**d**) collagen fiber assemblies of collagen fibrils; (**e**) bone mineral crystals, collagen molecules, and non-collagenous proteins. Image reproduced from Ref [81].

To fully understand the microstructure requirements of fully porous Ti implants, one must first understand the properties of human bone. Bone is essentially an open-cell composite material of fibrous protein, collagen, and calcium phosphate crystals, with an intricate vascular system forming various structures and systems in a five-level hierarchically organised structure [84,144]. According to Rho, et al. [145], these five hierarchical levels are dimensional scales ranging from the macro to the sub-nano levels (Figure 3).

Bone’s macrostructure is composed of cancellous and cortical bone, which are two different regions of bone with different density. Cortical bone forms the outside layer of the bone providing a strong, compact structure, leaving only 3–10% of the volume for its biological elements, such as blood vessels, osteocytes, erosion cavities, and canaliculi [81]. Cancellous bone forms the inside of the bone and is spacious and highly porous [81]. The pores are filled with bone marrow and the spacious architecture allows space for metabolic waste and nutrients to flow. Cancellous bone has an active metabolism and regenerates quicker compared with cortical bone [146]. By changing its density, cancellous bone can reorganise its structure depending on the stress direction [107]. These features cause cancellous bone mechanical properties to vary from bone to bone and change longitudinally [144].

The microstructure of bone ranges from 10 to 500 µm and it contains three major cavities. These are Haversian canals, osteocyte, lacunae, and canaliculi [88]. Cortical bone microstructure is composed of cylindrical structures called osteons with diameters ranging between 70 to 140 µm [81,87,92]. Along osteons’ central axis are pores called haversian systems, with diameters ranging between 20 to 50 µm, containing blood vessels and nerves [92]. Cancellous bone micro-architecture is composed of irregular units called trabeculae which create its porous structure. The pore geometry and pore size of cancellous bone porous structure is critical for cell distribution and cell migration [81]. Cancellous bone is naturally stochastic, with random pore distribution of pores of different size.

The pores in cancellous bone are ellipsoidal in the natural direction of loading and are usually 300–600 µm wide [147]. The interconnectivity between the pores of cancellous bone is essential for nutrient and waste diffusion.

**Figure 4:**
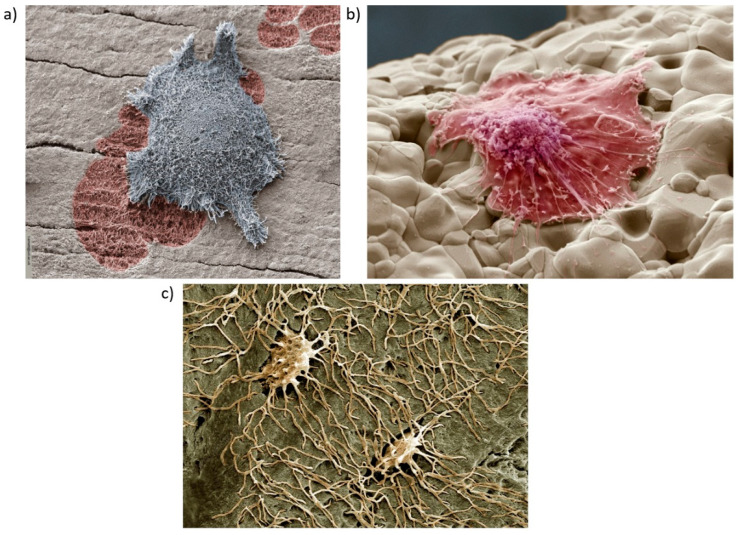
Coloured scanning electron micrographs of bone cells: (**a**) Activated osteoclast and resorption pit by kind permission of Timothy Arnett Ref [153]; (**b**) Osteoblast growing on a bone scaffold made of calcium oxide and silicon dioxide with added strontium and zinc by kind permission of Guocheng Wang from [154]; (**c**) Osteocytes embedded in the bone matrix with long cytoplasmic extensions reaching into the bone tissue, by kind permission of Kevin Mackenzie. Here, the minerals in the bone have been removed by embedding in resin and etching with perchloric acid. This reveals the spaces in the bone and the shape of the osteocyte cells.

At the micro level are also located the three types of differentiated bone cells osteoclasts, osteoblasts, and osteocytes, as shown in Figure 4. Osteoclasts and osteoblasts are vital for the functions of developing and healing bone tissue [86]. Osteoclasts (Figure 4a) are the main cells responsible for resorption of old bone tissue. Osteoblasts (Figure 4b) are bone cells responsible for bone formation, remodelling, fracture healing (for which they are critical), and bone development [148]. Osteocytes (Figure 4c) are osteoblasts cells present inside mature bone and serve as mechanosensory cells to control the activity of osteoclasts and osteoblasts [149].

Bone’s sub-microstructure, from 1 to 10 µm, is composed of lamellae which in cortical bone compromise the concentric layers of osteons and in cancellous bone lamellae forms the trabeculae volume [150,151]. The sub-nanostructure of bone, below a few hundred nanometres, is composed molecular constituent elements, such as collagen, non-collagenous organic proteins, and mineral. From a few hundred nanometres to 1 µm is bone’s nanostructure comprising fibrillar collagen with embedded hydroxyapatite nanocrystals [152].

#### 3.3.2. Bone Mechanical Properties

Most bones in the body are load bearing and require high mechanical strength. Bone tissue is anisotropic and stronger in compression than in tension. The mechanical properties must be measured in two orthogonal directions: longitudinal, which is the natural loading direction, and transverse [86]. The mechanical strength of bone is complex to measure as it varies with age, health, activity, and position in the body [86]. Bone becomes stiffer and less ductile with age and its ability to heal decreases [155]. It is also likely to weaken with immobilisation, such as for a person with movement disabilities or limited physical activity.

**Figure 5:**
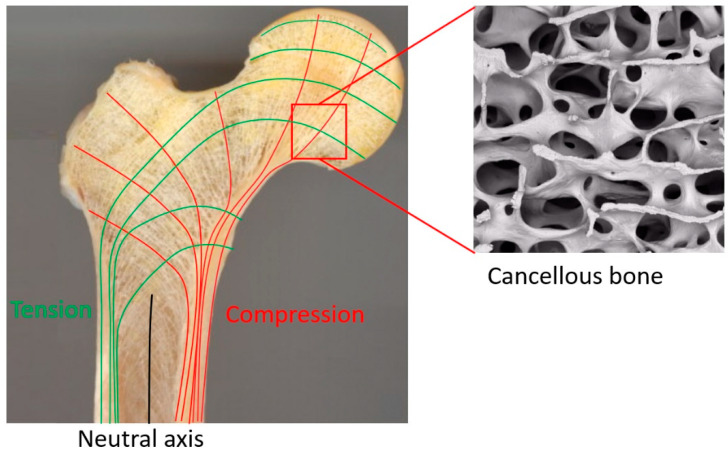
Cortical and cancellous bone; image adapted from [157,158] by kind permission of Alan Boyde.

The two different types of bone, cortical and cancellous, have completely different mechanical properties; therefore to specify the properties of bone as one material, both bone types must be considered. Cortical bone is highly dense an act like a shell that provides the greatest stiffness and resistance to bending. In contrast, the mechanical properties of cancellous bone are determined by its apparent density and trabecular architecture. The trabecular structure of cancellous bone is arranged accordingly to the stress distribution of load, as shown in Figure 5. As a result, the least material is used in the most strategic locations to carry the greatest loads with the least strain [156].

The mechanical properties of cortical and cancellous bone are difficult to measure, and tend to vary depending on bone orientation, test methods, mathematical formulas, and assumptions [146,151,159]. According to Chen and Thouas [57], the elastic modulus of cortical bone is approximately 11–21 GPa in the longitudinal direction and 5–13 GPa in the transverse direction. Another study suggested 18–22 GPa [147], whereas Wang et al. [81] suggested a range of 3–30 GPa. Cortical bone has a porosity of less than 10% [85]. The elastic modulus of cancellous bone is estimated to be 0.02–6 GPA and it has a high porosity of 50–90% [85,86].

To achieve adequate strength in bone implant design, one must understand the strength requirement of bone. Especially cortical bone must be considered, because the strength of such tissue is the minimum strength required by the implant. The compressive yield point of bone represents the threshold from where the structure accumulates irreversible deformation. Unlike metals such as steel, the yield point cannot be clearly distinguished, and it is rather associated with a continuous transition zone [160]. Furthermore, it has been proven that the compressive yield strength of bone varies depending on the anatomic site [161]. The challenges in determining the yield strength of cortical bone results in varying values in the literature. Researchers have estimated cortical bone to have a compressive yield strength of 133.6 ± 34.1 MPa [162]. The same characteristic was estimated to be 108–117 MPa by Yeni and Fyhrie [163]. Further studies have tested compressive yield strain using uniaxial compression and achieved 141.0 ± 5.0 [164], 111.0 ± 18.6 [165], 112.5 ± 9.5 [166], and 115.1 ± 16.4 MPa [167].

If the loading surpasses the yield point for bone, it will eventually reach the ultimate point. This point represents the ultimate compressive strength the bone can withstand until irreversible strains and damage occur. Past this point, macrocracks are formed and fracture occurs [160]. Unlike yield strength, the ultimate compressive strength of cortical bone can be exactly determined, using a stress– strain experiment [160]. However, due to bone properties differentiating, values vary in the literature. For example, Wang et al. [84] suggested that cortical bone has an ultimate compressive strength of 180–200 MPa, whereas Calori et al. [8] suggested a wider range of 130–290 MPa and Henkel et al. suggested 100–230 MPa [168]. Table 5 and Table 6 summarise the mechanical and dimensional properties of natural bone considered in this study.

### 3.4. Comparison of Properties of Porous Ti Scaffolds Fabricated by AM and Ideal Quality Attributes

#### 3.4.1. Unit Cell Geometry

Metamaterials can be rationally designed by changing their geometry at the microscale of the constituting unit cells of the porous structure. In this systematic search, a total of 169 porous scaffolds were identified as a rationally designed and fabricated for bone implant applications. It was found that there are three preferred strategies for fabricating bio-metamaterials: beam-based, sheet-based, and including irregular porous structures [172].

**Figure 6:**
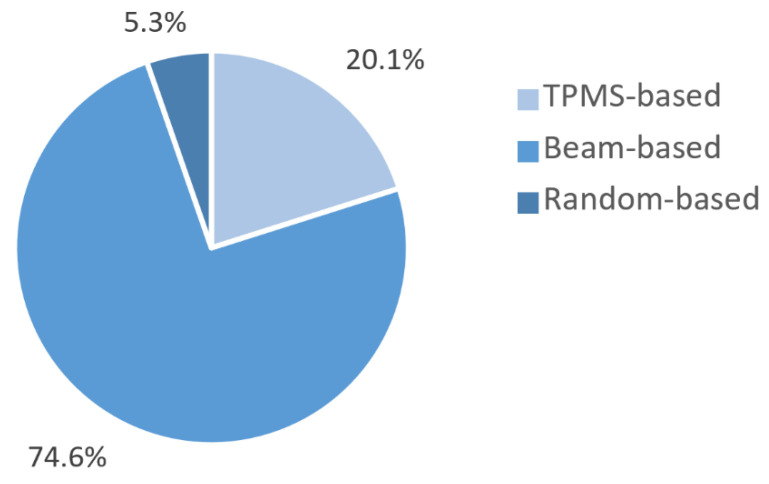
Distribution of experimental studies on beam-based, triply periodic minimal surfaces (TPMS), and random based geometries for bone implant applications.

According to Figure 6, the preferred design approach was beam-based, which represent 74.6% of the total scaffolds produced in the selected studies. The beam-based bio-metamaterials’ micro-architecture is composed of a lattice structure created using unit cells based on platonic solids, Archimedean solids, prisms and anti-prisms, and Archimedean duals [173,174,175,176,177] to mimic bone porous macro structure and mechanical properties such as modulus of elasticity. Nevertheless, the biological performance of bio-metamaterials created with beam-based geometries is limited by their inaccurate description of complex natural shapes due to their straight edges and sharp turns [109].

The unit cells of sheet-based geometries, on the other hand, are based on triply periodic minimal surfaces (TPMS), which are present in different organisms and cellular structures [178]. Therefore, it is no surprising that the second most used design strategy identified in this systematic search was sheet-based representing 20.1% of the total scaffolds produced, from which all used a TPMS as unit cell. Bio-metamaterials based on TPMS can mimic the various properties of bone to an unprecedented level of multi-physics detail in terms of mechanical properties and transport properties [79,172]. Moreover, the bone-mimicking mean surface curvature of zero of TPMS eliminates the effect of stress concentrators at nodal points [120].

In the case of irregular porous structures, are created in a random way to generate irregular porous structures that mimic trabecular bone geometry and mechanical properties [179]. These irregular structures are generated using the Voronoi and Delaunay tessellation methods. Irregular structures have been found to further enhance scaffold’s permeability and bone ingrowth compared with porous structures designed with regular unit cells [180]. According to our results only 5.3% of the studies used a randomised design approach to create irregular porous structures to mimic trabecular bone. This result was surprising considering that irregular porous structures were the first additively manufactured coatings used for orthopaedic implants in the medical industry. However, unlike random porous scaffolds, the great advantage of using regular repeating arrays of unit cells made of beam or sheet-based geometries is that they allow the creation of metamaterials with properties that can accurately be predicted [181]. This explain why these design strategies are preferred in research.

**Figure 7:**
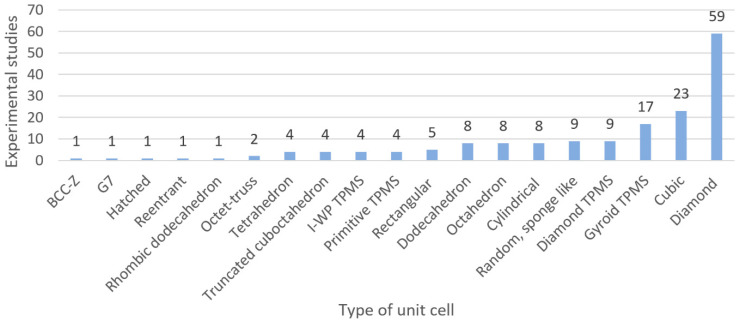
Comparison of the number of experimental studies of each unit used for bone regeneration identified in the reviewed articles.

**Figure 8:**
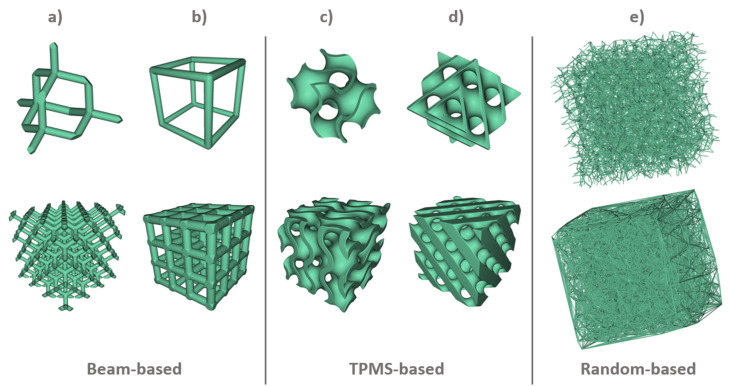
Some examples of unit cells with their corresponding meta-biomaterial scaffold below: (**a**) diamond beam-based unit cell; (**b**) cubic beam-based unit cell; (**c**) Gyroid triply periodic minimal surface based (TPMS-based) unit cell; (**d**) Diamond TPMS-based unit cell; and (**e**) Voronoi (top) and Delaunay (bottom) irregular porous structures.

From all the different possible unit cells that can be used to produce metamaterials, a total of 17 types of unit cell were used by the selected studies, as presented in Figure 7. According to Figure 7, the beam-based diamond unit cell (59 studies) was the most used, followed by the cubic (18 studies), and the gyroid TPMS (17 studies). These results correlate with the opinion of different experts who have stated that the diamond unit cell is the most studied for the development of metamaterials due to its biomimetic mechanical properties [121,182]. The high mechanical properties and self-supporting properties of the diamond unit cell are due to its unique geometrical arrangement, where one node is tetrahedrally surrounded by four other nodes coming from the crystal structure of the diamond crystal [113], as shown in Figure 8a. Moreover, this arrangement gives 48 symmetry elements to the diamond structure, making this unit cell invariant to different symmetry operations such as translations, reflections, rotations, and inversion [183]. Similarly, extensive research for bone regeneration has been performed to study the cubic unit cell (Figure 8b). The research interest in the cubic unit cell is because it is based on one of the simplest and easy–to–manufacture platonic solids thanks to its struts at an angle of 90° [62]. In the case of porous metamaterials based on the gyroid TPMS (Figure 8c), they have been found to exhibit similar topology to human trabecular bone, and also superior mechanical properties compared with metamaterials based on other types of TPMS [120]. For example, according to Yang et al. [184] metamaterials based on the gyroid TPMS have a more homogeneous stress distribution, which can provide equal mechanical stimulation to bone cells.

In the case of random or stochastic structures, Kou et al. [185] suggested that scaffolds based on this structures are more realistic; that is, they look more like natural bone with random non-uniform pore distribution and pore size [185]. Such structures are believed to provide benefits such as improved mechanical properties, including strength, fluid dynamics, surface area, and surface-to-weight ratio. They combine advantages of small and large pores without necessarily decreasing the mechanical strength or reducing the bone in-growth to levels that are inappropriate in application [186]. Figure 8 presents the five most representative unit cells identified in this study.

#### 3.4.2. Porosity

It is known that the degree of micro-porosity in bone implants directly affects their biological and mechanical properties. The porosity of natural bone is crucial for vascularisation, diffusion of cell nutrients and metabolic waste, and cell migration [187], and in a similar way it is important for metal bone implants. Moreover, several studies have considered porosity as the main parameter affecting stiffness and strength of porous biomaterials. Increased porosity reduces the strength of the implant [187,188]. As a result, porous metallic biomaterials are used as coatings in many medical applications, but more recently porous biomaterials have been proposed for load-bearing applications beyond surface coatings [138,142]. Ti and Ti alloys are commonly used for load-bearing implant applications due to their relatively low elastic modulus fatigue resistance, high strength to weight ratio, and corrosion resistance [82,94]. However, bulk Ti and Ti alloys do not completely match all the mechanical properties of natural bone such as modulus of elasticity. Therefore, it is a need of the hour to accomplish specific mechanical properties for Ti or Ti-based alloys by controlling the porosity and pore characteristics for customised implants [131]. However, the ideal porosity for medical implants seems controversial in the literature [181].

**Figure 9:**
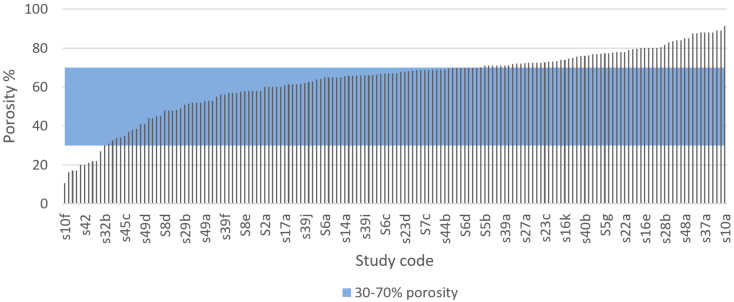
Porosity of implant specimens in the reviewed articles, compared with the Ideal Quality Attribute (IQA) target zone.

In this systematic review a total of 49 articles out of 50 recorded porosity of various degrees, as shown in Figure 9. For example, Stamp et al. [186] recommend using a porosity above 65% in medical implants whereas Ghanaati et al. [189] found that vascularisation increased in vivo when reducing the porosity from 80 to 40%. Sarhadi et al. [190] and Schiefer et al. [191] have recommended using a porosity of approximately 50%. According to Will et al. [192], the porosity that best promotes vascularization in porous scaffolds is 40–60%. Pattanayak et al. [193] manufactured porous Ti implants and reported an increase in compressive strength from 35 MPa to 120 MPa when reducing the porosity from 75 to 55%. Murr et al. reduced the porosity from 88 to 59% with an increase in stiffness from 0.58 GPa to 1.03 GPa [131]. As mentioned, natural cancellous bone has a porosity of 50–90% [85,86].

Zou et al. [102] designed three implants with similar pore size and shape. By reducing the porosity from 72 to 53% they achieved a compressive strength of 200 MPa and Young’s modulus of 4.3 GPa, instead of a compressive strength of 60 MPa and Young’s modulus of 2 GPa. Even though both implants achieved a stiffness close to that of human bone, only the implant with 53% porosity achieved a compressive strength greater than human bone. Hence, the implant with 72% porosity would not qualify as a load-bearing bone implant. These results, along with previous research by Pattanyak et al. and Murr et al., confirm the influence of porosity on mechanical properties [193]. They also show that the implant porosity may be adjusted within limits to increase strength and adjust bone stiffness, which varies for each patient’s characteristics.

From a medical regulatory perspective, the U.S Food and Drug Administration (FDA) only approves implant porosities of 30–70% for porous coatings on solid Ti implants [194]. The range is relatively large but can be used to one’s advantage since both the elastic modulus and strength of the implant can be adjusted by adjusting the porosity. Implants with porosity outside of this range do not comply with FDA regulations and cannot enter the market. It is known that medical regulations, especially for implantable medical devices, are based on strong scientific evidence. Therefore, it is vital to adhere to these regulations when designing bone implants.

Taking into consideration the medical regulations for porous implants, we selected a porosity range of 30–70% as the IQA for porous metal implants to identify studies in the systematic search that fabricated Ti scaffolds with porosity values within this range. From the 49 articles that recorded porosity of various degrees, a total of 167 results were extracted and compared with the selected IQA porosity range. The results of this comparison are presented in Figure 9. According to our results, 56.6% of the porous scaffolds studied in the 49 selected articles had porosity values within the acceptable porosity range (30–70%) required to satisfy medical regulations such as the FDA. By contrast, a total of 26 studies explored the properties of porous scaffolds with porosity values above the acceptable porosity range representing 37.3% of the total results extracted in this systematic literature review. There were several reasons for these studies to explore porosity levels higher than 70%. For example, Zhang et al. [100] and Amin Yavari et al. [106] fabricated different porous structures with various porosities to explore their mechanical properties and deformation mechanisms. Moreover, porous Ti scaffolds with high levels of porosity can serve as storage for mesenchymal stem cells to facilitate bone tissue regrowth, and also to improve cell oxygenation and nutrition [100]. On the other hand, Ti porous scaffolds with porosity levels lower than 30% can provide similar mechanical properties to cortical bone [98,100].

#### 3.4.3. Macropore Size

Since macro pore size is directly related to the strength, porosity, and stiffness of the implant, it is an important property for implant design [195]. Pore size has a profound effect on the behaviour of osteogenic cells even in an organ culture system [196]. The implant’s macro porosity determines whether bone cells can successfully penetrate and grow within the structure, and many studies have discussed the influence of pore size on the biological properties of implants [197]. Furthermore, several studies have shown that a minimum pore size of 100 µm is required for vascularization and bone ingrowth, but pores larger than 100 µm increase bone in-growth by allowing improved vascularization and oxygenation [86,91,193]. A minimum macropore size limit of 100 µm is supported by further research as vascular penetration has been found to be restricted in smaller pore interconnections [85,168,188,192].

Studies have found that pores greater than 300 µm are required for vascularisation and bone ingrowth [86,168]. Tang et al. [188] found that 200–350 µm is the optimal macropore size, and various studies have found that bone ingrowth is less likely to occur beyond 400 µm [90,195,198]. However, research that used pore sizes of 300, 600, and 900 µm in porous Ti scaffolds found that those with macropores sizes of 600 and 900 µm had much higher bone ingrowth compared with the scaffolds with 300 µm pores [101]. Bose et al. [85] suggested that all macropore sizes between 100 and 600 µm are osteoconductive. Fukuda et al. [199] experienced greater results in 500 and 600 µm pores compared with 900 and 1200 µm pores. Xue et al.’s [98] results showed that macropore sizes in the range of 100–600 μm possess the optimum ability for cell growth into the pore structure of porous titanium.

According to the FDA regulations, macropore sizes of 100–1000 um are approved for coatings for Ti implants [194]. Large macropores have a smaller surface area than do small pores, decreasing the cell attachment on the implant [86]. However, large macropores increase scaffold vascularisation, which is vital for supplying oxygen and nutrients to the tissue as well as osteoblast proliferation and migration [80], but they decrease the mechanical strength of the material. The limit of how much the macropore size can be increased while maintaining sufficient mechanical strength depends on both the material and the processing conditions. Therefore, regulatory guidelines for surface coating may be misleading for fully macroporous implants, since the strength-to-weight ratio differs between a porous and solid structure. Since the porosity decreases the strength of the implant, and large pore sizes decrease the strength of the internal architecture, large pore sizes must be avoided to increase the structure’s strength. A more defined pore size range is therefore sought [86].

In this study, the macroporosity used in different studies was explored. It was found that no consensus currently exists on what upper limit to macropore size that is ideal, but somewhat of a consensus on the lower limit exists (100 µm). According to FDA regulations, porous implants should have macropore sizes between 100 and 1000 µm [194]. It has further been found that macropores start to lose their osteogenic functionality when larger than 500–600 µm [8,16,85,98,199]. Considering that a fully porous structure is weaker than a solid structure, and that high strength is vital for implants, it can be assumed that there is no need to design a structure with pores larger than what is needed to cater for all functions within the implant.

**Figure 10:**
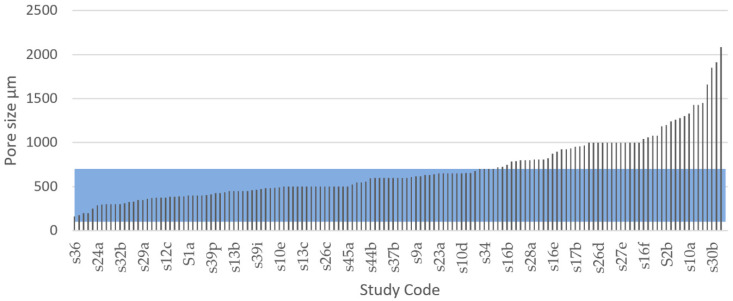
The pore sizes recorded in the reviewed articles, compared with the Ideal Quality Attribute IQA target zone.

These findings made us choose a macropore size range of 100–600 µm as the IQA for porous metal implants to identify the studies in the systematic search that fabricated Ti scaffolds with pore size values within this range. From the 42 articles that recorded pore size of various degrees, a total of 144 results were extracted and compared with the selected IQA pore size range, as seen in Figure 10. According to our results, 51.4% of the results of all studies had a macropore size within 100–600 µm. It was further noted that 86.8% of the experimental results of all studies had a macropore size within the FDA recommended range of 100–1000 µm. From these results, we could infer that most of the research studies identified through the systematic search somewhat considered the macroporosity range required to satisfy medical regulations.

By contrast, a total of 68 results out 144 showed pore size values above the acceptable macropore size range representing 48.6% of the total results extracted in this systematic literature review. There were several reasons for these studies to explore pore sizes above 600 µm. For example, the FDA approves macropore sizes between 100 and 1000 µm [194]. Hara et al. [96] tested four porous structures with different macropore size to explore their mechanical properties. Taniguchi tested 300, 600, and 900 µm pore sizes and found that the structures with 600 and 900 µm pore size exhibited higher bone ingrowth. Large macropores increase scaffold vascularisation, which is vital for supplying oxygen and nutrients to the tissue as well as osteoblast proliferation and migration [80]; however, larger pores also have a smaller surface area compared with small pores, decreasing the cell attachment on the implant [86]. Larger macropores also result in higher porosity, and this reduces the strength of the implant [187,188].

The ideal macropore size for bone implants is controversial and undefined, and according to Otsuki et al., a reason for the varied pore size data may be that the interconnectivity of pores was not considered [198]. Macropore size regulations have been developed for the first generation of porous implants, which use a single-scaled porous network with repeated, equally sized, and shaped pores. However, bone grows in a naturally random structure with pores of different sizes, shapes, and directions similar to the structure of a sponge [130,155]. The use of a multiscale porous scaffold that combines smaller and larger pore sizes within the same structure is a recent strategy to optimise the internal architecture of implants [142,188]. This method combines advantages of both small and large macropores without decreasing the strength or reducing the bone in-growth to levels that are inappropriate in application [188]. According to our results, a total of 8 out of 50 studies used some sort of multiscale pore approach, but it was observed that the design method varied, and that the researchers failed to provide the percentage of the total structure that used each pore size. It was further observed that a multiscale porous structure occurred in some implants where the manufacturing of a single-scaled structure resulted in varying pore sizes due to unprecise manufacturing tolerances.

#### 3.4.4. Pore Inter Connectivity

According to our results, 46% of the collected studies registered pore interconnectivity. Interestingly, all of these studies designed their porous scaffolds with an interconnectivity of 100%. The pores in a porous bone implant must be interconnected to ensure movement and the supply of necessary nutrients through ingrowth of tissue and bone [200]. Interconnected pores tend to facilitate the flow of fluids and biological cells through the structure which is essential for bone tissue formation [185]. According to Nyberg et al. [201] the integration of artificial material tissue with native tissue can be improved by interconnected pores. Tang et al. [188] suggested that an increased pore interconnectivity increases the number and size of blood vessels formed in scaffolds. The interconnectivity is also a critical factor for ensuring that all cells within the structure are within a 200 µm range from a blood supply to provide transfer of nutrients and oxygen [202]. According to the FDA’s recommendations for porous metal coatings, pores in such structures must be interconnected [194]. Although this requirement is for surface coatings, it also indicates the importance of an interconnected porosity for fully porous implants. In the systematic review, it appears as though a vast majority of studies had indicated the importance of an interconnected porosity. Therefore, to guarantee all processes and fluid movements necessary for tissue and bone ingrowth, the selected IQA for pore interconnectivity would ideally be 100%.

#### 3.4.5. Elastic Modulus

It was observed in this systematic review that an elastic modulus is a property commonly reported in AM porous scaffolds studies (by 89% of all studies). A controlled modulus of elasticity has proved to be critical in prostheses and scaffolds to avoid stress shielding [81,82]. Stress shielding occurs when there is a stiffness mismatch between the implant and surrounding bone, and it can cause inflammation and the need for revision surgery [197]. Ti and common implant Ti alloys have an elastic modulus of roughly 100–120 GPa [81,84,138,197]. A reduced modulus is necessary to avoid stress shielding and can be achieved by designing implants with a porous structure [90].

Defining an ideal specific modulus of elasticity for porous bone implants is not practical because Since the mechanical properties of human bone, especially the elastic modulus, change drastically with factors, such as age, physical activity, and health. For example, femoral bone specimens from patients aged 3, 5, and 35 years had an elastic modulus of 7, 12.8, and 16.7 GPa, respectively, indicating a dramatic change with age [90]. As previously shown in Table 6, the elastic modulus of human bone varies in the literature. Chen and Thouas [57] estimated the elastic modulus of cortical bone to be approximately 11–21 GPa in the longitudinal direction, whereas Lee et al. [147] suggested 18–22 GPa. Wang et al. [81] suggested a wider range of 3–30 GPa. These findings indicate that the stiffness of an implant may need to be adjusted specifically for the person it is intended for, and that the target value for the elastic modulus may be specific to each patient. Therefore, it is more practical to think that for the design of porous scaffolds, an ideal target area exists for the modulus of elasticity. Based on this, the IQA for elastic modulus is proposed to be 3–30 GPa for fully porous Ti implants.

**Figure 11:**
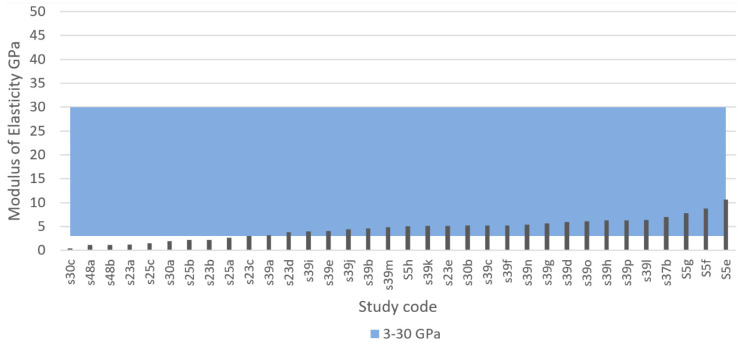
Elastic modulus of porous metamaterials based on triply periodic minimal surfaces (TPMS) compared with the Ideal Quality Attribute (IQA) target zone.

**Figure 12:**
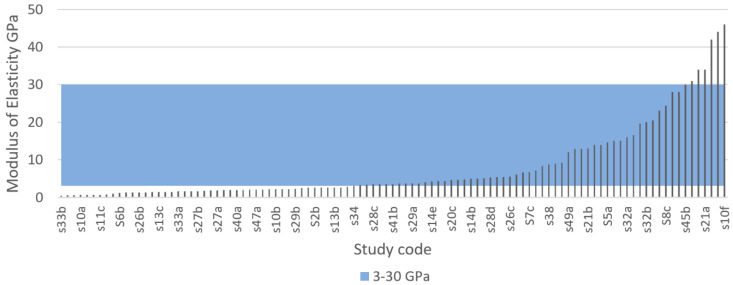
Elastic modulus of porous beam-based metamaterials compared with the Ideal Quality Attribute (IQA) target zone.

Figure 11 and Figure 12 show the elastic modulus that was reported in the reviewed articles and these values were compared with the proposed IQA. From the extracted of elastic modulus results, 55.5% reached the target area of 3–30 GPa. These implants achieved an elastic modulus within the range of natural bone and would therefore eliminate risk of stress shielding. By contrast, 40% of the results exhibited an elastic modulus below 3 GPa, and only 3.6% of the results reported an elastic modulus higher than 30 GPa. These results clearly demonstrated that most studies are aiming towards a modulus of elasticity closer to the bone modulus.

The elastic modulus of metals such as titanium and its alloys naturally have a much higher elastic modulus compared with bone [81]. However, research shows that the elastic modulus of metals can be readily adjusted by modifying their porosity. Porous metals with a low modulus of elasticity correspond to high levels of porosity. For example, Wang et al. [84] explored five types of porous structures using the same material (TiNbZr) and pore size (550 µm) but ranging porosity. His results revealed that four of the implants with porosities ranging from 42% to 69% all achieved an elastic modulus within the approved range of 3–30 GPa; however, the implant with the highest porosity (74%) achieved the lowest elastic modulus of 1.6 GPa. Similarly, Li et al. [203] used a porosity of 91% resulting in a low elastic modulus of 0.8 GPa. Furthermore, Chen et al. [204] received an elastic modulus of 44.4 GPa for a porous titanium structure using 30% porosity but by increasing the porosity to 40% the elastic modulus was reduced to 24.7 GPa.

**Figure 13:**
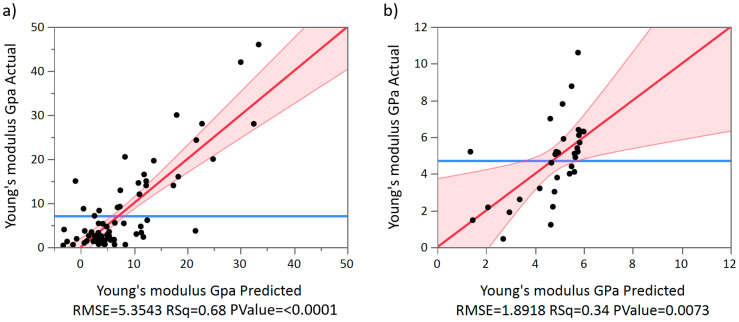
Overall predicted model of elastic modulus actual versus an elastic modulus predicted (**a**) beam-based scaffolds, and (**b**) TPMS-based scaffolds.

Using the data obtained through the systematic literature search we calculated two multiple linear regressions to predict the modulus of elasticity of beam and TPMS-based AMd Ti scaffolds. The regression model used the independent variables of pore size, relative density (porosity), and the interaction of pore size–porosity. According to our results, a regression equation was found for beam-based AMd Ti scaffolds (F(3,75) = 54.139, *p* < 0.0001), with an R^2^ adj of 0.671, as shown in Figure 13a. The residuals of the multiple linear regression are randomly scattered around the centre line of zero with no obvious pattern. The predicted compressive yield strength of beam-based scaffolds is equal to 27.738 – 0.078 (pore size) – 27.417 (porosity) + 0.0689 (pore size*porosity), where pore size is coded or measured in µm, and relative density expressed as porosity as a percentage. The beam-based scaffolds’ modulus of elasticity decreased 0.078 MPa for each µm, 27.417 MPa per 1% of porosity increment, and increased 0.0689 MPa for the interaction pore size*porosity. Both pore size (*p* < 0.0005) and porosity (*p* < 0.0001) were significant predictors of beam-based scaffolds’ modulus of elasticity, including the interaction between pore size and porosity (*p* < 0.0001).

In the case of the multiple linear regression of TPMS based AMd Ti scaffolds a significant regression equation was also found (F(3,28) = 4.897, *p* < 0.0073), with an R^2^ adj of 0.273, as shown in Figure 13b. The residuals of the multiple linear regression are randomly scattered around the centre line of zero with no obvious pattern. The scaffolds’ predicted modulus of elasticity is equal to 0.008 − 0.002 (pore size)–2.342 (porosity) + 0.002 (pore size*porosity). The TPMS based scaffolds’ modulus of elasticity decreased 0.002 MPa for each µm, 2.342 MPa per 1% of porosity increment, and 0.002 MPa for the interaction pore size*porosity. Pore size was a significant predictor of TPMS-based scaffolds’ modulus of elasticity with a *p*-values < 0.0563. However, porosity was not a significant predictor with *p*-values < 0.553 and 0.843, respectively. Moreover, no interaction between pore size and porosity was found regarding to modulus of the elasticity.

#### 3.4.6. Compressive Yield Strength

For an adequate functioning of any load-bearing implant, it is vital that its design withstand the required forces and loading cycles. Mechanical strength is one of the implant’s most crucial features for avoiding implant failure. To withstand the loads of daily activities, load-bearing implants must have at least the same yield strength as the bone that they replace [81]. The yield point of bone represents the threshold from where the structure accumulates irreversible deformation. Unlike bulk metals such as steel, the yield point of bones cannot be clearly distinguished; it is rather associated with a continuous transition zone [160]. Strain beyond the yield point will deform the structure beyond its point of resilience causing material damage, usually occurring as micro-cracks [205]. Bone tissue has evolved to mainly support compressive stress [206,207]. Bone is 30% weaker under tensile stress, and 65% weaker under shear stress [208]. Therefore, load-bearing implant scaffolds require a high compressive strength to prevent fractures and improve functional stability [209]. The compressive yield strength of cortical bone varies in the literature. As previously shown in Table 6, the compressive yield strength of cortical bone varies approximately between 90 and 170 MPa. To replace like with like, using a biomimetic approach for comparison purposes, a minimum and a maximum compressive yield strength of 90 MPa and 170 MPa were selected as the IQA for fully porous Ti implants.

**Figure 14:**
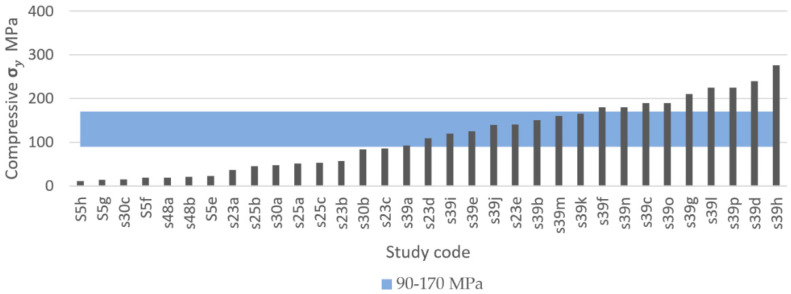
Compressive yield strength of porous metamaterials based on triply periodic minimal surfaces (TPMS) compared with the Ideal Quality Attribute (IQA) target zone.

**Figure 15:**
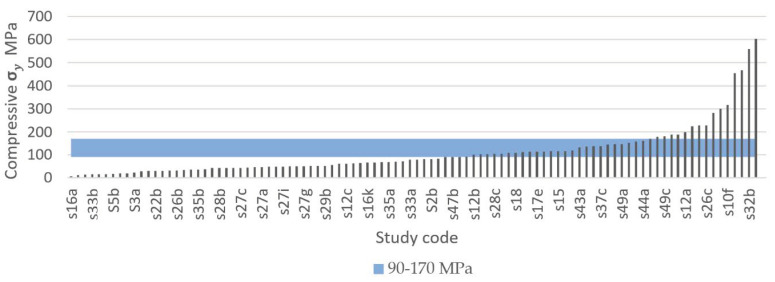
Compressive yield strength of porous beam-based metamaterials compared with the Ideal Quality Attribute (IQA) target zone.

The systematic search identified that 37 out of 50 studies recorded compressive yield strength, from which a total of 133 experimental results were extracted and compared, as shown in Figure 14 and Figure 15. Figure 14 presents the results of studies using TPMS structures and Figure 15 presents the results of studies using porous beam-based metamaterials. Both comparisons show high numbers of studies resulting in a compressive yield strength below 90 MPa. A total of 55.7% of all results had a compressive yield strength below the defined IQA target and 25% of the studies achieved a compressive yield strength within the bone region. On the other hand, only 19% of the extracted experimental results had a strength above the bone region. Such implants would have strengths similar to or higher than cortical bone and are expected to not experience permanent deformation caused by the expected bone compressive loading conditions in the human body.

Decreased strength of a porous implant can result from high porosity and large pore sizes, [81,188]. For example, Zhang et al. [113] fabricated porous scaffolds based on the TPMS diamond unit cell with a wide range of compressive yield strengths from 36 MPa to 140 MPa just by varying the scaffolds’ porosity and maintaining the pore size constant. The type of unit cell used to design porous scaffolds can also drastically change it mechanical properties. For example, Zhao et al. [116] fabricated four porous scaffolds with the same pore size and similar porosities using two different unit cells (tetrahedron and octahedron). However, the scaffolds based on the octahedron unit cell registered almost double the compressive strength compared with those based on the tetrahedron unit cell [116]. The compressive yield strength of porous metals can also be enhanced by gradually changing the porosity level along the radial direction of the scaffold. This was demonstrated by Zhang et al. [100], who reported functionally graded porous scaffolds based on the diamond unit cell with superior comprehensive mechanical properties to the biomaterials with uniform porous structures.

Using the data obtained through the systematic literature search, we calculated two multiple linear regressions to predict compressive yield strength based on pore size, porosity, and the interaction of size–porosity for beam-based and TPMS-based AMd Ti scaffolds, respectively.

**Figure 16:**
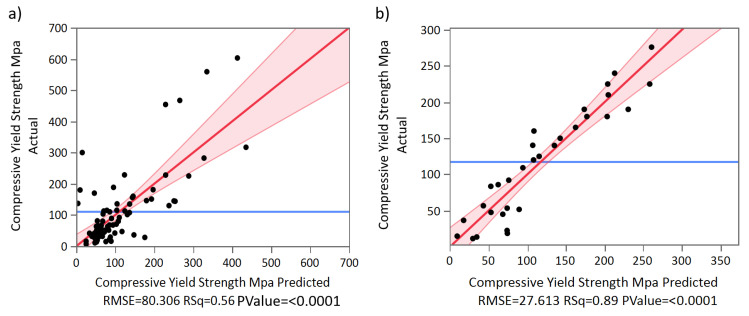
Overall predicted model of compressive yield strength Actual versus compressive yield strength Predicted of (**a**) beam-based scaffolds, and (**b**) TPMS-based scaffolds.

According to our results, a regression equation was found for beam-based AMd Ti scaffolds (F(3,75) = 31.452, *p* < 0.0001), with an R^2^ adj of 0.539, as shown in Figure 16a. The residuals of the multiple linear regression are randomly scattered around the centre line of zero, with no obvious pattern. The scaffolds’ predicted compressive yield strength is equal to 380.557 − 0.075(pore size)–350.828 (porosity) + 0.557 (pore size*porosity), where pore size is coded or measured in µm, and porosity is measured as a percentage. The beam-based scaffolds’ compressive yield strength decreased 0.075 MPa for each µm, 350.828 MPa per 1% of porosity increment, and increased 0.557 MPa for the interaction of pore size*porosity. Both pore size (*p* < 0.0378), porosity (*p* < 0.001), and the interaction between pore size and porosity (*p* < 0.0048) were significant predictors of beam-based scaffolds’ compressive yield strength. The interaction between pore size and porosity was found to be significant with a *p*-value < 0.0048.

Regarding the multiple linear regression of TPMS-based AMd Ti scaffolds, a significant regression equation was also found (F(3,27) = 65.547, *p* < 0.0001), with an R^2^ adj of 0.872, as shown in Figure 16b. The residuals of the multiple linear regression are randomly scattered around the centre line of zero, with no obvious pattern. The scaffolds’ predicted compressive yield strength is equal to 524.780 − 0.008(pore size)–625.266 (porosity) + 0.183 (pore size*porosity). The TPMS-based scaffolds’ compressive yield strength decreased 0.008 MPa for each µm, 625.266 MPa per each 1% of porosity increment, and increased 0.183 MPa for the interaction of pore size*porosity. Porosity was a significant predictor of TPMS based scaffolds’ compressive yield strength with *p*-values < 0.0001. However, pore size and the interaction between pore size and porosity were non-significant, with *p*-values < 0.7018 and 0.3260, respectively.

#### 3.4.7. Ultimate Compressive Strength

If loading surpasses the yield point of bone, it will eventually reach the ultimate point. This point represents the maximum compressive strength that a material can withstand without irreversible strains and damage occurring. Past this point, macrocracks are formed and fracture occurs [160]. Bone implants in load-bearing applications must withstand high stress within the body, to a degree where no permanent deformation occurs during the load that the implant is expected to be exposed to. Hence, controlled ultimate compressive strength is a crucial property to study in bone implant research. Natural bone is estimated to have an ultimate compressive strength of 180–200 MPa [84]. However, results vary in the literature. For example, Calori et al. [8] suggested a more widespread range of 130–290 MPa, whereas Henkel et al. suggested 100–230 MPa [168]. To replace like with like using a biometric approach, bone implants should have an ultimate compressive strength similar to that of bone [8]. Taking into consideration the compressive yield strength suggested previously and the three results presented in Table 5, the proposed IQA region for the ultimate compressive strength is between 180 MPa and 290 MPa.

**Figure 17:**
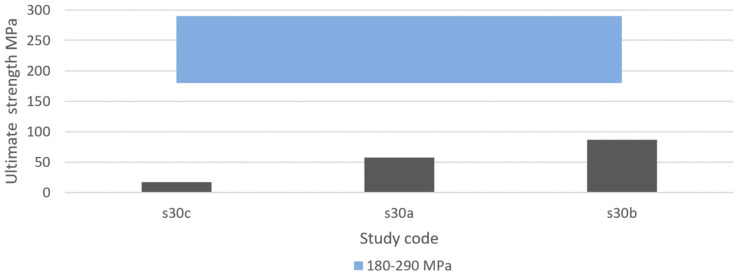
Ultimate compressive strength of porous metamaterials based on triply periodic minimal surfaces (TPMS) compared with the Ideal Quality Attribute (IQA) target zone.

**Figure 18:**
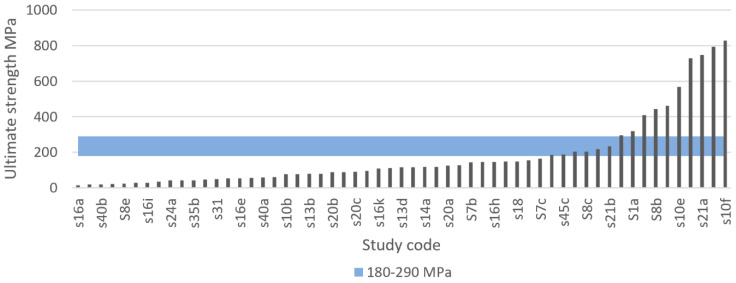
Ultimate compressive strength of porous beam-based metamaterials compared with the Ideal Quality Attribute (IQA) target zone.

In this systematic search a total of 60 experimental results of ultimate compressive strength from 19 different studies were extracted. Figure 17 and Figure 18 show the ultimate compressive strength of the different studies compared with the defined IQA target of between 180 MPa and 290MPa. Figure 17 corresponds to experimental results of porous scaffolds composed of TPMS unit cells compared with the IQA target. According to Figure 17, only one study with three different experimental results measured the ultimate strength of porous scaffolds based on TPMS unit cells. In this study by Yanez et al. [120], three ultimate compressive strengths of 17, 47.5, and 83.5 MPa were achieved using the gyroid unit cell. Dramatic improvement in the ultimate compressive strength of the scaffold was achieved. This improvement in mechanical properties was possible by slightly changing the gyroid unit cell into an elongated gyroid. However, none of the experimental results obtained by Yanez et al. [120] were able to reach the minimum IQA ultimate compressive strength proposed in this study. The low strength of Yanez at al.’s [120] samples can be attributed to their high porosity values which ranged between 75% and 90%. This increased the stress concentration, reducing the ultimate compressive strength.

Figure 18 is composed of 19 different studies on beam-based unit cells where 57 experimental results are compared with the IQA target. According to Figure 18, 10 experimental results reached higher ultimate compressive strengths than bone. The highest ultimate compressive strength (830 MPa) recorded was achieved with a scaffold based on the diamond unit cell by Zhang et al. [100]. From all results recorded, a total of 16.7% achieved an ultimate compressive strength above the proposed IQA ultimate compressive strength; 10% had similar ultimate compressive strengths to bone; and 73.3% had lower ultimate compressive strengths than bone. The scaffolds that did not fulfil the required IQA ultimate compressive strength would risk fracturing due to macrocracks occurring during high loads. Porosity within a structure has been proven to decrease the strength of a structure [81,188], which may explain the high number of scaffolds with low ultimate strength.

The goal of designing a porous structure with enough porosity and pore size without diminishing strength is a difficult task, and as researchers aim to create highly porous structures with low elastic modulus, many structures experienced low ultimate strength. Attar et al. [111] manufactured a porous titanium structure by SLM with rectangular pores and 17% porosity and achieved an ultimate compressive strength of 747 MPa. Using the same material, manufacturing method, and unit cell, a different structure with 37% porosity achieved an ultimate strength of 235 MPa. In a similar manner, Chen et al. [204] designed three structures of the same material and manufacturing method. Using porosities of 30%, 40%, and 50%, the ultimate compressive strengths recorded were 524, 301.7, and 120.3 MPa, respectively, indicating that increased porosity reduces the strength of the structure.

#### 3.4.8. Fatigue Strength

During normal daily activities, load-bearing implants experience just a fraction of the material’s ultimate stress [209,210,211]. However, after years of use, the high cyclic loading to which load-bearing implants are subjected eventually leads to the accumulation of small stresses, causing progressive and localised material damage that results in implant failure [212]. For instance, one of the most critical mechanical properties for load-bearing implants is fatigue strength. However, fatigue strength is the most difficult mechanical property to determine [213].

The required fatigue resistance of a load bearing implant and its components mainly depends on their cyclic loading conditions and the required life span. For example, it is estimated that lower limb prostheses are subjected to up to 2 million gait cycles per year [214], and in the case of orthodontic prostheses these can reach up to 300,000 loading cycles per year [213]. Therefore, a large variety of medical standards exist for testing fatigue strength. Some of these fatigue tests differ depending on the type of load applied such as tension–tension, compression–compression, and tension–compression. In the case of load-bearing bones, their loading conditions in real-life activities are complex [135]. However, bone is mainly loaded in compression [206,207]. Therefore, to test the fatigue life of metamaterials for bone implant applications, compression fatigue tests are preferred due to the simplicity of the test setups [215].

Regarding the number of cycles that load bearing implants and their components need to have tested for fatigue strength, all the different medical standards agree that such products need to have a fatigue life within the high-cycle fatigue region (N > 10^4^ cycles). For example, the ASTM standard F2777 – 16 recommends testing tibial inserts’ endurance and deformation under high flexion with a minimum number of cycles of 2.2 × 10^5^, and in the case of dental implants they are typically tested up to 5 million cycles [213,216]. Nevertheless, for a component of a load-bearing implant to have at least 25 years of life span [217], the highest number of cycles that must be tested is 10^7^ cycles [213,216]. Taking into consideration current medical standards for load-bearing implants, the high-cycle fatigue region between 10^4^ cycles and 10^7^ cycles was selected as the IQA fatigue life for porous titanium metamaterials for bone regeneration.

**Figure 19:**
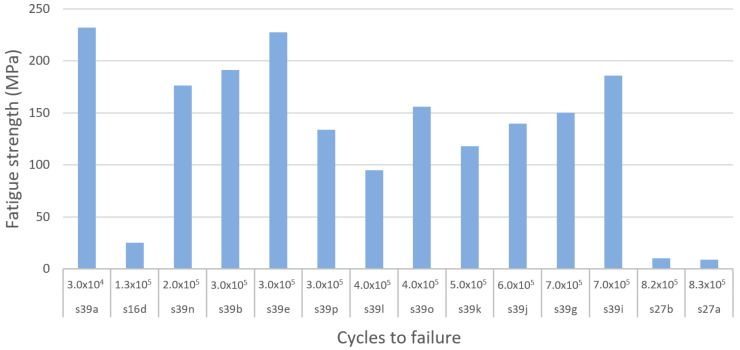
Comparison of the results of different studies on the fatigue strength of porous metamaterials based on triply periodic minimal surfaces (TPMS) within the high-cycle fatigue region between 10^4^ and 10^5^ cycles.

**Figure 20:**
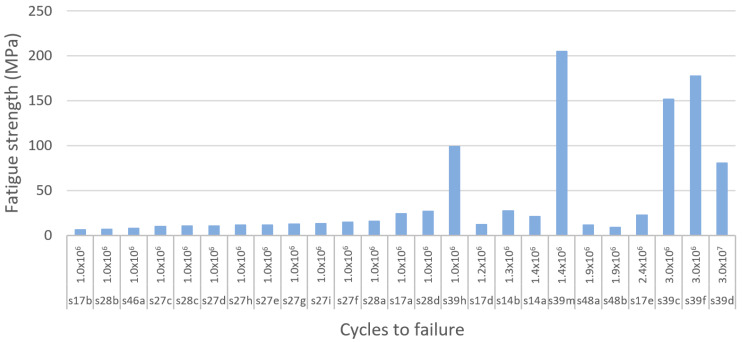
Comparison of the results of different studies on the fatigue strength of porous metamaterials based on triply periodic minimal surfaces (TPMS) within the high-cycle fatigue region between 10^6^ and 10^7^ cycles.

**Figure 21:**
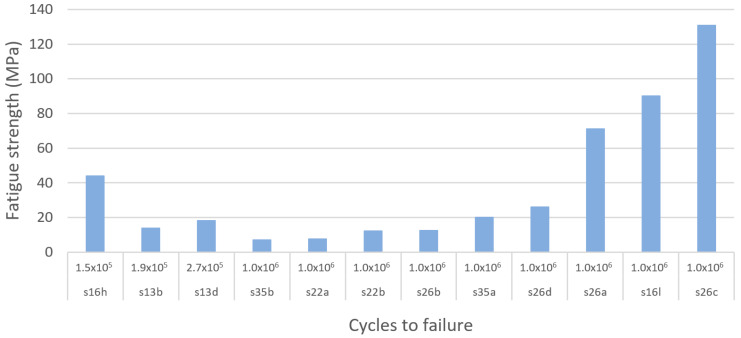
Comparison of the results of different studies on the fatigue strength of porous beam-based metamaterials within the high-cycle fatigue region between 10^6^ and 10^7^ cycles.

Using the selected high-cycle fatigue region, this systematic literature search identified a total of 13 different studies on fatigue resistance, among which 11 studies performed compression–compression fatigue tests. Then, for comparison purposes, a total of 51 experimental results were extracted and compared. Moreover, to facilitate the comparison of the results of the studies, they were classified according to the type of unit cell used to produce porous structures as beam and TPMS-based as resented in Figure 19, Figure 20 and Figure 21. According to our results, the TPMS porous structures that withstood the highest stresses at the high-cycle fatigue region were achieved by Bobbert et al. [79]. The primitive TPMS structure presented the highest stress within the high-cycle fatigue region, with 232 MPa at 3 × 10^4^ cycles, as shown in Figure 19. The TPMS porous structures that were able to withstand the second and third highest stresses within the high-cycle fatigue region were the I-WP and diamond structures, with 227 MPa at 3 × 10^5^ cycles and 204 MPa at 3 × 10^6^ cycles as shown in Figure 19 and Figure 20. Remarkably, the primitive TPMS structure was the only one to pass the 10^7^ threshold with 80 MPa at 3 × 10^7^ cycles, as presented in Figure 20.

In the case of beam-based metamaterials the study performed by Zhao et al. [116] achieved the highest fatigue strength with 130 MPa at 10^6^ cycles using a lattice structure based on the octahedron unit cell, as shown in Figure 21. In second and third place with the highest fatigue strength are the tetrahedron and the cubic porous lattice structures with 90 MPa at 1 × 10^6^ cycles and 90 MPa at 1 × 10^6^ cycles fabricated by Amin Yavari et al. [106] and Zhao et al. [116], as presented in Figure 21.

According to the results of this systematic search it could be seen that TPMS structures provide superior fatigue strength to porous bio-metamaterials compared with beam-based unit cells. Moreover, in terms of fatigue resistance, it was identified that the primitive, I-WP, and diamond TPMS provided the best performance, whereas the octahedron, cubic, and tetrahedron are the best-performing lattice unit cells. However, it is crucial to note that several factors can affect the fatigue life of additively manufactured Ti metamaterials. Some of these factors are residual stresses and stress concentrators caused by high surface roughness and manufacturing defects [218]. Moreover, the fatigue strength of bulk materials significantly degrades when they are in porous form or when voids and pores are developed during fabrication [219]. In the case of additively manufactured components, it has been proven that their fatigue strength is extremely sensitive to localised and nonuniform heat and uncontrolled cooling cycles during fabrication [220,221].

### 3.5. Discussion and Summary of the Findings

Table 7 presents a summary of proposed IQA target values for porous Ti and Ti alloy bone implants aimed at load-bearing applications. These values are based on scientifically supported values found in human bone research, federal regulations (FDA), and research articles on porous Ti implants manufactured using AM between 2000 and 2020. These properties are part of the first step of the QbD framework of “Define the Quality Target Product Profile.” The IQAs are necessary for a systematic and qualitative design approach. These properties will provide benchmark guidance to facilitate future research on porous bone implant design.

An IQA target zone for the porosity of porous Ti implants has been proposed as 30–70% based on results found in research articles as well as in current FDA regulations. We found that 56.6% of all studies in this review achieved a porosity within this range. AM was found to produce porous structures with highly controlled porosity. Numerous studies as well as FDA regulations have discussed the importance of using porous structures for tissue ingrowth, which have numerous advantages to non-porous implants. Whereas porous coatings are used in some instances, it was observed in this study that many researchers believe in using a fully porous structure to achieve a biomimetic structure mimicking natural bone. All data used extracted in this systematic literature review came from studies that used scaffolds with constant porosity. However whether a repeated lattice structure throughout the entire structure is sufficient or a biomimetic “sponge-like” structure with irregular, elongated pores should be used has not yet been confirmed in research; nevertheless implants using a biomimetic design approach replicating natural bone received attention in recent research [86]. Since bone has a random and stochastic structure with pores of different sizes, a multiscale porous structure with pores of different sizes deserves further research. Multiscale porous scaffold structures have been proven to perform better than one-dimensional structures [142]. Nonetheless, to properly compare implants with a multiscale structure, pore distribution and size must be recorded.

In the case of scaffolds’ pore size, the IQA was proposed to be 100–600 µm based on numerous performed research trials. Whereas the FDA requirement is a pore size between 100–1000 µm, a more defined pore size range of 100–600 µm is supported by research due to numerous research trials having experienced reduced bone ingrowth in pores larger than 600 µm. Strut thickness is the thickness of the pore walls within a porous structure. It may have a substantial impact on implant mechanical properties [90] and can serve as a unit cell’s characteristic to compare different mechanical properties in relation to it. However, it was found that struct thickness is not commonly reported in the literature.

Research has proven that the elastic modulus of bone changes drastically with age and that patient customisation is a necessity in specific cases. As a result, in this study, an IQA for elastic modulus was proposed to be 3–30 GPa based on values from research studies. According to data analysed in this study a porous structure has a significant influence on mechanical properties in Ti-based implants. Therefore, a porous structure can be altered to provide an elastic modulus comparable to that of natural bone and be adjusted to modify the elastic modulus according to the patient’s age and health condition. This will further reduce the risk of stress shielding between the implant and surrounding tissue.

**Figure 22:**
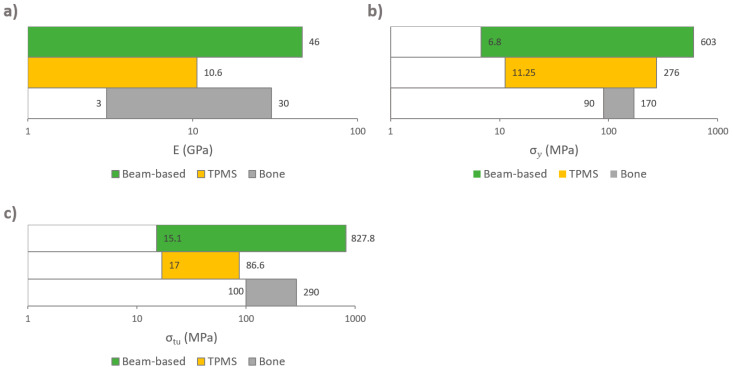
Comparison in logarithmic scale between mechanical properties of bone, beam-based, and triply periodic minimal surfaces (TPMS): (**a**) modulus of elasticity; (**b**) compressive yield strength; and (**c**) ultimate compressive strength.

As reported by most studies it was found in the regression analysis that the elastic modulus of Ti and its alloys is directly influenced by the implant porosity, and that by increasing the porosity the elastic modulus is increased and vice versa. A total of 55.5% of the studies in this review recorded an elastic modulus within this range. Regarding the effect of unit cells on the modulus of elasticity of porous structures, it was found that the studies that used beam-based unit cells covered a wider range of modulus of elasticity values than did those than used TPMS-based unit cells (Figure 22a). Moreover, the studies that used beam-based unit cells also obtained modulus of elasticity values within the whole range of human bone Young’s modulus, as shown in Figure 22a.

Compressive yield and ultimate strength were studied. The compressive strength values for cortical bone were found to vary in the literature, which is why a range of values was selected to represent this bone characteristic. The IQA for compressive yield strength was proposed to be a minimum of 90 MPa and the IQA target for ultimate compressive strength a minimum of 180 MPa. A total of 44.1% of the studies achieved a compressive yield strength above 90 MPa, whereas only 26.7% achieved an ultimate compressive strength above 180 MPa. Both TPMS-based and beam-based scaffolds provided a wide range of compressive yield strengths covering the whole range of human bone compressive yield strength, as presented in Figure 22b. However, according to Figure 22c only beam-based scaffolds provided an adequate range of ultimate compressive yield strength values that include bone properties. The narrow range of ultimate compressive yield strength values of TPMS-based scaffolds can be explained by the lower number of studies that have addressed this mechanical with this type of unit cell. This indicates that a need exists for TPMS-based scaffold research to report the ultimate compressive yield strength.

Just as with the elastic modulus, the strength of implants was found to be directly affected by scaffold porosity. Increased porosity was found to rather dramatically decrease the strength of the implant as demonstrated by the regression model performed with the results of several different studies. Moreover, it was found that large pores are directly related to lowered strength and elastic modulus, and that several studies have struggled to produce highly porous structures with strength higher than or close to natural bone. Therefore, to ensure the adequate strength of implants, pores larger than required (>600 µm) and porosity values higher than necessary (>70%) are recommended to be avoided as these have been found to likely result in low-strength structures.

## 4. Conclusions

This systematic literature review presented an overview of the reported properties in research studies of fully porous Ti bone implants manufactured with AM received in the last two decades. The study focused on implants made of Ti and Ti alloy since they are considered ideal biomaterials for load-bearing applications. This study followed a QbD approach and includes the first step of the QbD system that defines the QTPP for properties relating to the porous internal architecture of fully porous Ti implants designed for load bearing applications. IQA, part of the QbD system, have been proposed supported by properties of natural human bone, governmental regulations, and scientific research relevant to bone implants. Unit cell geometry, porosity, elastic modulus, compressive yield strength, ultimate compressive yield strength, and compressive fatigue strength were systematically reviewed and benchmarked against the proposed IQA.

This study found that many implant geometrical, mechanical, and dimensional characteristics were directly related to each other. Scaffolds’ pore size influences the porosity of the structure, and the porosity alters the elastic modulus as well as the strength of the implant. The unit cell geometry was also found to directly affect the Young’s modulus and strength of porous scaffolds, and naturally would also impact the structures’ interconnectivity. Moreover, by using ranges rather than set values where possible, such as for elastic modulus, porosity, and pore size, there is a flexibility to the design that allows the implant to be adjusted to its purpose and patient. Such design flexibility is necessary as bone properties vary with patients’ age and anatomic site.

Despite the variety of scaffold characteristics reviewed in this study, future systematic literature searches should also focus on other properties, such as fluid dynamics, surface finish (topography), creep, and hardness, as well as surface coatings. Moreover, it should be considered that bone ingrowth may modify implants’ mechanical properties; hence, they may be dependent on the level of bone ingrowth. Therefore, it is important to measure the changes in strength and stiffness in metallic bone scaffolds after bone ingrowth has occurred. Since implant location may determine the importance of each implant property, future studies would ideally find IQA for different implant locations within the human body. For example, mechanical features are important to study in load bearing implants, whereas fluid dynamics and vascularisation might be more important features for facial implants that require good aesthetic results and that are placed close to fragile tissue and nerves.

This study was possible due to the abundance of data available in research articles. However, to further develop an effective QbD engineering strategy for the development of specific bone implants it is important to identify the degree of importance of each bone scaffold characteristic according to the implant’s future location within the human body. This can be identified by following the second step in the QbD system, namely Critical Quality Attributes (CQA), as detailed described in previous work [222]. CQA are product characteristics that must fall within specific limits to comply with the quality standards defined in the QTPP. They can be identified through prior knowledge and experimental data from systematic research based on scientific and risk management rationale that considers regulatory and business requirements [223]. Each step of the QbD system must be further developed to facilitate the strategic, qualitative development of bone implants and increase the rate of successful research studies.

Overall, Ti and Ti alloy porous bone implants are well underway to achieving the ideal properties that will fully allow them to replace natural bone. With the help of the QbD system, consistent and qualitative design of medical implant devices can be achieved.

## Figures and Tables

**Table 1:** Search strategy, studies between 2000 and 2019.

Database	Records Identified	Total
Google Scholar	3020	5941
Science Direct	2921	
Duplicates	80	5861

**Table 2:** Studies selected in the systematic search according to information related to bone properties.

Ref	Cod.	Pore Characteristics				Mechanical Properties		
		Pore Size	Pore Shape	Porosity	Interconnectivity	Young’s Modulus	Yield Strength	Compressive Strength
[79]	S51	✔	✔	✔	✔	✔	✔	
[80]	S52			✔		✔	✔	✔
[81]	S53	✔	✔	✔	✔	✔	✔	✔
[82]	S54			✔	✔	✔	✔	✔
[83]	S55					✔		
[84]	S56							✔
[85]	S57			✔	✔	✔		
[86]	S58			✔	✔			
[87]	S59	✔						
[88]	S60	✔	✔	✔	✔			
[89]	S8	✔		✔	✔	✔		
[90]	S62			✔		✔		
[91]	S63					✔	✔	✔
[84]	S64	✔		✔		✔		
[92]	S65	✔						
[93]	S66					✔	✔	✔

**Table 3:** Studies selected in the systematic search according to information related to additively manufactured (AMd) porous Ti structures’ mechanical, geometrical, and dimensional properties.

Ref	Cod.	Pore Characteristics					Mechanical Properties			
		Size	Unit Cell Geometry	Porosity%	Connectivity	Multi-scaled	Young’s Modulus	Compressive Yield Strength	Ultimate Compressive Strength	Fatigue
[19]	S1	✔	✔	✔	✔		✔		✔	
[94]	S2	✔	✔	✔			✔	✔		
[82]	S3	✔	✔	✔	✔		✔	✔	✔	
[95]	S4	✔	✔			✔	✔	✔		✔
[62]	S5	✔	✔	✔	✔		✔	✔		
[96]	S6	✔	✔	✔			✔			
[97]	S7		✔	✔			✔		✔	
[98]	S8	✔	✔	✔	✔		✔		✔	
[99]	S9	✔	✔	✔	✔					
[100]	S10	✔	✔	✔	✔	✔	✔	✔	✔	
[101]	S11	✔	✔	✔			✔	✔		
[102]	S12	✔	✔	✔	✔	✔	✔	✔		
[103]	S13	✔	✔	✔	✔		✔	✔	✔	✔
[104]	S14		✔	✔	✔		✔	✔	✔	✔
[105]	S15	✔	✔	✔				✔		
[106]	S16	✔	✔	✔	✔			✔	✔	✔
[107]	S17	✔	✔	✔	✔			✔	✔	✔
[108]	S18	✔	✔	✔	✔		✔	✔	✔	
[109]	S19	✔	✔	✔	✔	✔				
[110]	S20	✔	✔	✔		✔	✔		✔	
[111]	S21		✔	✔	✔		✔	✔	✔	
[112]	S22	✔	✔	✔			✔	✔		✔
[113]	S23	✔	✔	✔	✔		✔	✔		
[114]	S24	✔	✔	✔	✔				✔	
[115]	S25	✔	✔	✔			✔	✔		
[116]	S26	✔	✔	✔	✔		✔	✔		✔
[117]	S27	✔	✔	✔			✔	✔		✔
[118]	S28	✔	✔	✔			✔	✔		✔
[119]	S29	✔	✔	✔		✔	✔	✔		
[120]	S30	✔	✔	✔			✔	✔	✔	
[121]	S31	✔	✔	✔	✔	✔	✔	✔	✔	
[122]	S32	✔	✔	✔	✔		✔	✔		
[123]	S33	✔	✔	✔	✔	✔	✔	✔		
[124]	S34	✔	✔	✔	✔		✔		✔	
[125]	S35	✔	✔	✔			✔	✔	✔	✔
[126]	S36	✔	✔	✔			✔		✔	
[127]	S37	✔	✔	✔			✔	✔		
[128]	S38	✔	✔	✔	✔		✔	✔	✔	
[79]	S39	✔	✔	✔			✔	✔		✔
[129]	S40	✔		✔			✔		✔	
[130]	S41	✔	✔	✔	✔		✔			
[131]	S42	✔	✔	✔			✔	✔		
[132]	S43	✔	✔	✔	✔			✔		
[133]	S44	✔	✔	✔	✔		✔	✔		
[134]	S45	✔	✔	✔			✔		✔	
[135]	S46		✔	✔						✔
[136]	S47	✔	✔	✔	✔	✔	✔	✔		
[137]	S48		✔	✔			✔	✔		✔
[138]	S49	✔	✔	✔			✔	✔		
[139]	S50	✔	✔	✔			✔	✔		

**Table 4:** The ideal quality dimensions of porous internal architecture of Ti bone implants.

Quality Approach	Dimension	Description
**Product-based approach**	Performance	The porous microstructure should provide an environment ideal for bone ingrowth and endow the implant with a stiffness similar to natural human bone while maintaining sufficient strength.
	Features	Tailored internal architecture with specific properties, including but not limited to pore size, unit cell, porosity, elastic modulus, interconnectivity, compressive yield, and ultimate strength, as well as fatigue strength.
	Reliability	Optimised fabrication of porous Ti structures with high mechanical strength as well as a high degree of reproducibility, minimal defects, and zero failure rates (within their life expectancy).
**Manufacturing-based approach**	Manufacturability	The scaffold’s micro-geometry should be designed in such a way that it is easy to manufacture with high accuracy and definition.
	Conformance	The mechanical, geometrical, and dimensional characteristics should comply with medical regulations and quality standards.
	Durability	Porous Ti structures should withstand mechanical forces experienced during handling, implantation surgery, and operation thereafter in a traumatised bone microenvironment constantly under load.
**User-based approach**	Perceived quality	Clinicians should have access to relevant characteristics through medical reports and statistical data where implant performance can be seen.

**Table 5:** Summary of dimensional properties of natural human bone.

Material	Pore Size	Pore Shape	Porosity	Interconnectivity	Ref
**Cancellous bone**	300–600 µm	Spongy, ellipsoidal pores	50–90%	55–70%	[82,84,89,91,169]
**Cortical bone**	10–50 µm	Cylindrical canals	3–10%	-	

**Table 6:** Summary of mechanical properties of natural human bone.

Material	Young’s Modulus	Compressive Yield Strength	Ultimate Compressive Strength	Compression Fatigue Strength	Ref
**Cancellous bone**	0.02–6 GPa	7.2–23.2 MPa	17–33 MPa	72.6–124 MPa at 10^6^ cycles	[8,82,84,89,91,93,162,165,168,169,170,171]
**Cortical bone**	3–30 GPa	92.4–167.7 MPa	100–290 MPa	137 MPa at 10^6^ cycles	

**Table 7:** Summary of Ideal Quality Attributes proposed in this study.

Ideal Quality Attributes	
Porosity	30–70%
Pore size	100–600 µm
Elastic modulus	3–30 GPa
Compressive yield strength	90–170 MPa
Ultimate compressive strength	180–290 MPa
Fatigue resistance	72.6–137 MPa at 106
Interconnectivity	100%
